# Adjustments in physiological and morphological traits suggest drought‐induced competitive release of some California plants

**DOI:** 10.1002/ece3.8773

**Published:** 2022-04-01

**Authors:** Justin C. Luong, Michael E. Loik

**Affiliations:** ^1^ Environmental Studies Department University of California, Santa Cruz Santa Cruz California USA

**Keywords:** competitive release, environmental filter, intrinsic water‐use efficiency (*i*WUE), optimal partitioning, percent loss of net assimilation (PLA), δ^13^C

## Abstract

Drought and competition affect how morphological and physiological traits are expressed in plants. California plants were previously found to respond less negatively to resource limitation compared to invasive counterparts. In a glasshouse in Santa Cruz, CA, USA, we exposed five native California C_3_ grassland species to episodic drought and competition (via five locally invasive species). We hypothesized that leaf morphology would be more affected by competition, and leaf photosynthetic gas exchange more so by drought, consistent with optimal partitioning and environmental filter theories. We expected that traits would exhibit trade‐offs along a spectrum for resource conservatism *versus* acquisition. *Bromus carinatus* had greater photosynthetic recovery, while *Diplacus aurantiacus* had lower percent loss of net assimilation (PLA) and intrinsic water‐use efficiency (*i*WUE) during drought and competition simultaneously compared to just drought. *Stipa pulchra* and *Sidalcea malviflora* gas exchange was unaffected by drought, and leaf morphology exhibited drought‐related adjustments. *Lupinus nanus* exhibited trait adjustments for competition but not drought. Functional traits sorted onto two principal components related to trade‐offs for resource conservatism *versus* acquisition, and for above‐ *versus* belowground allocation. In summary, morphological traits were affected by competition and drought, whereas physiological traits, like leaf gas exchange, were primarily affected by drought. The grassland plants we studied showed diverse responses to drought and competition with trait trade‐offs related to resource conservatism *versus* acquisition, and for above‐ *versus* belowground allocation consistent with optimal partitioning and environmental filter theories. *Diplacus aurantiacus* experienced competitive release based on greater *i*WUE and lower PLA when facing drought and competition.

## Graphical abstract

In a glasshouse in Santa Cruz, CA, USA, we exposed five native California C_3_ grassland species to episodic drought and competition (via five locally invasive species). In summary, morphological traits were affected by competition and drought, whereas physiological traits, like leaf gas exchange, were primarily affected by drought. The grassland plants we studied showed diverse responses to drought and competition with trait trade‐offs related to resource conservatism *versus* acquisition, and for above‐ *versus* belowground allocation consistent with optimal partitioning and environmental filter theories.

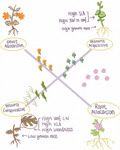

## 1 INTRODUCTION

Optimal partitioning theory suggests that plants increase biomass allocation to structures that acquire the most limiting resource (Bloom et al., 1985). Stressors can differently affect physiological and morphological traits. Physiological traits are those related to molecular‐level interactions of compounds within a plant, whereas morphological traits determine plant shape or structure (Lambers et al., 2008). Water‐limited plants have been shown to partition growth more so to root than shoot structures (Liu & Stützel, 2004). Biotic stressors such as competition can have more varied impacts because it unevenly interacts with abiotic resources, which is further complicated by species‐specific responses (Rehling et al., 2021). Invasive competition could lead to increased allocation to shoots or leaves to increase access to space and light (Pérez‐Harguindeguy et al., 2016; Westoby, 1998), or increased allocation to roots to access limiting belowground resources, especially in abiotically harsh systems (Liu & Stützel, 2004; Poorter et al., 2012).

Droughts can lead to shifts in the root‐to‐shoot ratio (root:shoot) or adjustments in leaf traits related to resource conservative plant strategies (Heckathorn & Delucia, 1996). Plants that are more resource conservative typically grow slower, use less resources, and are more drought resistant, while resource acquisitive species may be more resilient in their recovery from drought or grow fast during wet periods to escape drought (Funk et al., 2008; Kooyers, 2015). Different mixes of acquisitive and conservative traits allow some species to recover from drought (Nicotra et al., 2010), while others may experience unrecoverable physiological stress (Zhong et al., 2019). Photosynthetic rates and biomass allocation are often reduced by drought, and although some species may recover photosynthetic rates fully upon rewetting, others may not (Poorter et al., 2012; Zhong et al., 2019). Certain plants have higher water‐use efficiency (WUE) after drought (Lajtha & Marshall, 1994), whereas others have decreased WUE and lower photosynthetic recovery (Zhong et al., 2019) leading to feedbacks that can result in mortality.

Environmental filter theory (Funk et al., 2008) predicts that individuals have to pass through abiotic and biotic filters to establish or sustain co‐existing populations at a particular site (Adler et al., 2013). Abiotic filters like drought often result in different species having similar conservative traits to survive the same harsh micrometeorological conditions. On the other hand, biotic filters facilitate species trait divergence, partitioning of resources, and allowing for species coexistence (Poorter et al., 2012). Passing through abiotic and biotic filters at a particular site may require contrasting values of the same traits (Funk et al., 2008; Pierce et al., 2017). Harsh abiotic conditions and limited resource availability select for resource conservative traits like low specific leaf area (SLA), stomatal conductance (*g*_s_), and growth rates, whereas strong biotic filters associated with competition select for high net CO_2_ assimilation (*A*_net_), SLA, and high growth rates (Drenovsky et al., 2012; Pérez‐Harguindeguy et al., 2016). Leaf lobedness and vein length can promote trait conservatism by reducing leaf water loss (Cadotte et al., 2015; Sack & Scoffoni, 2013). California will likely have more frequent droughts and continued species invasions that may lead to trade‐offs that balance the selective pressures of opposing environmental filters (Ishida et al., 2008; Pierce et al., 2017; Seebens et al., 2015).

Strategies such as drought escape, avoidance, and tolerance are coordinated by physiological and morphological traits, and can be used to further understand plant responses to global change (Kooyers, 2015; Levitt, 1980). Drought tolerance and escape are more consistent with the classic leaf economic spectrum theory, while drought avoidance coordinates characteristics not typical of the leaf economic spectrum (Kooyers, 2015; Sandel et al., 2021; Volaire, 2018; Wright et al., 2004). Drought tolerance is more common for woody species with conservative traits (Ingram & Bartels, 1996; Volaire, 2018). Drought escape and avoidance are more common for herbaceous species with acquisitive traits that have active growth during periods of high soil water availability, distinct from drought‐tolerant species that can maintain growth during periods with low soil water (Huang et al., 2018; Kooyers, 2015; Welles & Funk, 2021). Drought escape is common for annuals and is typified by quick growth and high fecundity (Huang et al., 2018). Drought avoidance is prevalent for both annuals and perennials, and these species rely on high WUE, limited vegetative growth, and high root:shoot ratio (Kooyers, 2015; Levitt, 1980).

Competitive release results in increased fitness or productivity for a species when its competitor is removed or negatively affected by environmental conditions (Menge, 1976; Segre et al., 2016). California plants may experience competitive release during drought because their invasive counterparts respond more negatively to drought compared to native annuals in greenhouses and perennials *in situ* (Luong et al., 2021; Valliere et al., 2019). Certain native perennial bunchgrasses are able to withstand competition from invasive species (Corbin & D’Antonio, 2004), but less is known about other life‐forms. California species that are affected by invasion have lower aboveground productivity and some species adjust leaf traits associated with competitive ability to maximize fitness (Drenovsky et al., 2012; Seabloom et al., 2003). Yet, how invasive competition and drought interact to drive plant growth, morphology, and competitive release is less understood (Poorter et al., 2012; Segre et al., 2016).

We tested how drought and invasive competition shape functional traits and biomass allocation for five California grassland species commonly used for restoration in central California. In a controlled glasshouse environment in Santa Cruz, CA, USA, we measured physical traits (biomass, growth rates, specific leaf area, leaf area, major vein length per unit area, leaf lobedness, leaf C:N, and δ^13^C) and photosynthetic gas exchange rates (*A*_net_, *g*_s_) of native species experiencing episodic drought and invasive competition. Environmental filter theory predicts that plants will grow slower under drought, so we hypothesized droughted plants would have reduced instantaneous leaf‐level gas exchange, and also greater root allocation due to optimal partitioning. We predicted that competition would lead to changes in leaf traits to acquire space and light resources. We also hypothesized native species would exhibit trade‐offs that fall on a spectrum related to resource conservatism (high VLA, lobedness, *i*WUE, and C:N; see methods) versus acquisition (high SLA, ARGR, *A*_net_, and leaf N) observed via functional traits in response to factorial drought and competition, as predicted by the leaf economic spectrum and environmental filter theory.

## 2 MATERIALS AND METHODS

The five native species in this study were chosen because they are commonly used for grassland restoration in California (Table 1; Jepson eFlora, 2020). We selected the five invasive species (Table 1) based on their high cover from previous vegetation surveys (Luong et al., 2021). The invasive species are regionally ubiquitous and monitored by the California Invasive Plant Council (www.cal‐ipc.org). All seeds were sourced from experimentally restored areas at Younger Lagoon Reserve in Santa Cruz, CA, USA (36.951918°N, 122.063116°W; 7 m a.s.l.). Seeds were collected from multiple individuals on ambient rainfall (control) plots of a field drought experiment (Loik et al., 2019).

**TABLE 1:** Family, life‐forms, and origin of the experimental grassland species

Scientific name	Family	Life‐form	Origin
*Diplacus aurantiacus* Curtis.	Phrymaceae	Perennial semi‐woody shrub	Native
*Sidalcea malviflora* (DC.) A. Gray	Malvaceae	Perennial rhizomatous forb	Native
*Bromus carinatus* Hook. & Am.	Poaceae	Perennial bunchgrass	Native
*Stipa pulchra* Hitchc.	Poaceae	Perennial bunchgrass	Native
*Lupinus nanus* Benth.	Fabaceae	Annual N‐fixer	Native
*Medicago polymorpha* L.	Fabaceae	Annual N‐fixer	Invasive
*Festuca bromoides* L.	Poaceae	Annual grass	Invasive
*Carduus pycnocephalus* L.	Asteraceae	Annual forb	Invasive
*Raphanus sativus* L.	Brassicaceae	Annual forb	Invasive
*Geranium dissectum* L.	Geraniaceae	Annual forb	Invasive

### 2.1 Experimental design

We set up a two‐way factorial study manipulating drought and competition from invasive species in a rooftop glasshouse at the University of California, Santa Cruz, between October 2019 and April 2020. In October 2019, we sowed seeds of native species (Table 1) on PRO‐MIX high porosity soil (6:1:1 of sphagnum peat moss, perlite, and limestone) in seedling flats partitioned by species. Seedlings were kept well watered and then healthy seedlings similar in size from each species were individually transplanted into 32 4.5‐L growing containers (17 cm tall × 16 cm diameter). Transplanting occurred at least 2 weeks after germination and after plants developed two sets of true leaves. Once transplanted, the native plants were well watered and unfertilized for 6 weeks. Because most fertilizers are water based, droughted plants could not be fertilized, so all plants were kept unfertilized. We randomized pot locations on the glasshouse tables weekly to limit microclimate effects. Average daytime temperatures and relative humidity (RH) were 16.5°C and 68.1% while nocturnal conditions were an average of 10.7°C and 78.4% RH. Proportions of light‐to‐dark hours started at 11 h light to 13 h dark in October 2019, slowly decreased to its minimum in December, with 9.5 h light to 14.5 h dark, and increased to reach 13 h light to 11 h dark at the end of the study in April 2020. We did not augment the light intensity or cycle.

Eight replicates of each species were assigned to treatments within a 2 × 2 factorial design: (1) well watered (no manipulation); (2) episodic drought; (3) invasive competition; and (4) invasive competition and episodic drought simultaneously. We harvested three replicates from each native species in each treatment group to determine baseline aboveground and belowground biomass during week 6, leaving five replicates per species in each treatment.

On week 6 we sowed five common invasive species (Table 1) in half of all pots to establish the competition treatment. We sowed invasives at densities based on historic field surveys (Heady, 1977; 185 mg per pot *C*. *pycnocephalus*, 100 mg *F*. *bromoides*, 103 mg *G*. *dissectum*, 85 mg *M*. *polymorpha*, and 69 mg for *R*. *sativus*) corrected for the surface area of a 4.5‐L pot (201 cm^2^). On week 8, we applied an episodic drought (Duan et al., 2014) where water was withheld until a minimum stomatal conductance (*g*_s_; see list of abbreviations in Table 2) occurred for native species in an initial and secondary drought period (*g*_s_ <0.05 mol m^−2^ s^−1^ H_2_O). Rehydration occurred concurrently for all individuals of the same species after half of the individuals droughted from that species reached the minimum *g*_s_ threshold. The *g*_s_ was measured for all native individuals using an open‐mode portable photosynthesis system (Model LI‐6400; Li‐Cor, Inc.). Droughted plants were then rehydrated to pot capacity for 10 days, then exposed to a second drought. This episodic drought protocol with two drought periods has been shown to result in plant glasshouse drought responses that best mimic *in situ* plants (Duan et al., 2014). Due to interspecific variation in stomatal conductance to episodic drought (Table S1), the duration of drought varied for each native species. No native species had premature mortality. Non‐natives used for the competition treatment persisted through the drought to the end of the experimental period (Table S1).

**TABLE 2:** Glossary of commonly used eco‐physiological abbreviations

Abbreviation	Parameter
AGB	Aboveground biomass (g)
*A*_net_	Leaf net CO_2_ assimilation (µmol CO_2_ m^−2^s^−1^)
ARGR	Aboveground relative growth rate (g·day^−1^)
ARR	Net CO_2_ assimilation recovery rate (µmol CO_2_ m^−2^s^−1^ day^−1^)
BGB	Belowground biomass (g)
BRGR	Belowground relative growth rate (g·day^−1^)
C:N	Leaf carbon:nitrogen ratio (unitless)
*g*_s_	Leaf stomatal conductance (mol H_2_O m^−2^s^−1^)
*i*WUE	Intrinsic water‐use efficiency (µmol CO_2_ mol H_2_O^−1^)
PC	Principal component
PLA	Photosynthetic loss of net assimilation (%)
PRA	Photosynthetic recovery of net assimilation (%)
SLA	Specific leaf area (cm^2^·g^−1^)
VLA	Major vein length per unit area (cm^−1^)
WUE	Water‐use efficiency (µmol CO_2_ mol H_2_O^−1^)
δ^13^C	Carbon isotope fractionation (proxy for WUE, ‰)

During the second episodic drought, native plants were maintained under treatments until at least half of the plants in the drought treatment reached *g*_s_ < 0.05 mol H_2_O m^−2^ s^−1^. All individuals of that species were then harvested for final biomass measurements. The experimental period lasted 73–130 days depending on the species.

### 2.2 Functional traits

Traits were only sampled from native species. We collected three replicates of biomass from each species and treatment group prior to any treatments (week 6) and for all remaining individuals after the second episodic drought. We cut each plant at the base of the soil where the shoots and roots were differentiated. We washed soil out of the belowground biomass samples by gently dunking them in a series of four buckets with gentle agitation by hand. After the final bucket, we ran water over the roots to remove any remaining silt or perlite while over a 500 µm sieve to prevent root loss. We saved roots that broke off while washing to be included in dry biomass weights and estimated a loss of approximately 5% of total root biomass. Samples were dried at 60°C for at least 72 h before quantifying aboveground (AGB) and belowground biomass (BGB). We calculated aboveground relative growth rates (ARGR) and belowground relative growth rates (BRGR) by subtracting the final biomass of an individual by the baseline average taken in pretreatment (week 6), divided by the total growing days (Table 2).

We sampled leaves from native plants prior to any treatments and at the end of the second drought to quantify effects on specific leaf area (SLA), major vein length per unit area (VLA), leaf lobedness, leaf C:N, and δ^13^C (see list of abbreviations in Table 2). Pretreatment leaf characteristics and biomass were used to confirm there was no grouping effect prior to experimental treatments (*p*_all_ > .05). SLA is related to photosynthetic ability, palatability, leaf life span, and growth rates (Sandel et al., 2021; Wright et al., 2004). SLA often decreases in response to drought but increases due to competition (Wright et al., 2004). Total leaf area is associated with competitive ability because it is related to light capture, shading, water loss, and energy budgets (Liu & Stützel, 2004; Pérez‐Harguindeguy et al., 2016). Increased VLA can improve drought resistance by increasing vein reticulation and redundancy for water and sugar transport (Sack & Scoffoni, 2013). Leaf lobedness affects the leaf energy balance and is calculated as the ratio of leaf perimeter squared to the product of leaf area and *π* (Cadotte et al., 2015; Luong et al., 2021). Grass leaves may not be dissected but operationally, can have high leaf lobedness because of their high leaf perimeter:area ratios. Increased leaf lobedness decreases the effective length that wind travels at the leaf surface and reduces the boundary layer, resulting in increased cooling via conduction and convection, potentially decreasing leaf‐level transpiration (Lambers et al., 2008). Leaf C is related to palatability and leaf N to photosynthesis (Pérez‐Harguindeguy et al., 2016). Plants with high C:N values are often more resistant to drought but may be less competitive than plants with low leaf C:N (Drenovsky et al., 2012; Pérez‐Harguindeguy et al., 2016). δ^13^C is often used as a proxy for WUE (Table 2) because they are correlated for most species (Lajtha & Marshall, 1994).

We measured midday leaf gas exchange once prior to treatments, weekly during treatments (including the rewatering period), and once during dark hours (01:00 to 04:00 h) at the end of the second experimental drought period. For each species, midday measurements were conducted between 10:00 and 15:00 h. For each individual, we selected new but fully expanded leaves to use for gas exchange measurements, typically three levels below the apical meristem for cauline species. For bunchgrasses, we sampled leaves two levels outwards from the center and avoided leaves from flowering stalks. The order plants were measured were randomized weekly, so no treatment groups or individuals were consistently measured earlier or later in the day. We used a Model LI‐6400XT portable photosynthesis system for all gas exchange measurements. Inside the leaf chamber, photosynthetically active radiation (PAR; 400–700 nm) was set at 1500 µmol m^−2^ s^−1^, air temperature was 24°C, and CO_2_ concentration was 400 µmol mol^−1^. We started and calibrated measurements under identical glasshouse conditions (see above), took measurements only when the CV threshold was <0.2%, and acquired three instantaneous measurements at least 90 s apart to average for a certain leaf on a particular date. Intrinsic water‐use efficiency (*i*WUE) was calculated as the ratio of net CO_2_ assimilation (*A*_net_) to *g*_s_ (Table 2).

The resistance and resilience of leaf‐level photosynthesis (Zhong et al., 2019) were calculated as the percent loss of net assimilation (PLA; Equation 1) due to drought, and the percent recovery of net assimilation following rewatering (PRA; Equation 2). PLA and PRA are measured after the first drought period to provide a baseline for recovery after rehydration.

(1) $$\text{PLA}\left(\%\right)=\left(\frac{A_{i}‐A_{d}}{A_{i}}\right)\times 100\%$$

and

(2) $$\text{PRA}\left(\%\right)=\left(\frac{A_{r}}{A_{i}}\right)\times 100\%$$

*A*_i_, *A*_d_, and *A*_r_ represent *A*_net_ prior to drought, the end of the first drought period, and after rewatering, respectively. The assimilation recovery rate (ARR) is related to drought resilience and was calculated with Equation (3), where *D*_r_ represents the number of days between *A* measurements. Because these measurements require a drought period, they were only calculated for plants in the drought and not well‐watered treatments.

(3) $$\text{ARR}=\left(\frac{A_{r}‐A_{d}}{D_{r}}\right)$$

### 2.3 Analyses

All analyses were completed with R statistical software (Version 4.0.4; R Development Core Team, 2007). We ensured data had a Gaussian distribution and equal variances before using parametric tests. We used different statistical tests depending on the hypothesis to be tested. Data were processed and visualized with *plyr*, *cowplot*, and *ggplot2* (Wickham, 2020; Wickham et al., 2018; Wilke, 2020).

Because PLA, PRA, and ARR were only measured for individuals that experienced drought, the differences between droughted individuals with or without invasive competition were analyzed using *t*‐tests. Traits (SLA, VLA, lobedness, C:N, δ^13^C, and root:shoot biomass) collected at the end of the second drought period were compared using two‐way analysis of variance (ANOVA) to test for interactive effects of drought and invasive competition. Competitive release was defined on a physiological basis where there was greater *i*WUE, ARR, PRA, or lower PLA during combined drought and competition, compared to when plants were exposed to drought with no competition (Segre et al., 2016). For data collected weekly (*A*_net_, *g*_s_, and *i*WUE), we used mixed linear models with time as a fixed variable to test for the effects of drought and competition over time. We used a regression to test for a correlation between δ^13^C and *i*WUE.

We used a principal component analysis (PCA) to detect trade‐offs between measured traits along a spectrum of two principal components (PC) using the *vegan* package (Ishida et al., 2008; Oksanen et al., 2018; Pierce et al., 2017). PCA can be used to decrease dimensionality in multivariate trait space by compressing multiple variables into fewer selected intercorrelated axes (principal components). Trait values were then tested for correlations against main PCs to determine intertrait relationships (Pierce et al., 2017; Table S2). Related traits are summarized into a singular PC with positively correlated traits on one end of the axis and negatively correlated traits along a diametrically opposed vector. Individual species (experimental units) plot near the traits for which they have high values on the PCA (Pierce et al., 2017). Within this study, the resulting ordination provides a first approximation of trade‐offs between below‐ and aboveground growth (optimal partitioning) as well as resource and conservative traits (filter theory). Traits were categorized based on descriptions from Pérez‐Harguindeguy et al. (2016). Funk et al. (2008), Sack and Scoffini (2013), and Poorter et al. (2012).

## 3 RESULTS

### 3.1 Growth responses

**FIGURE 1:**
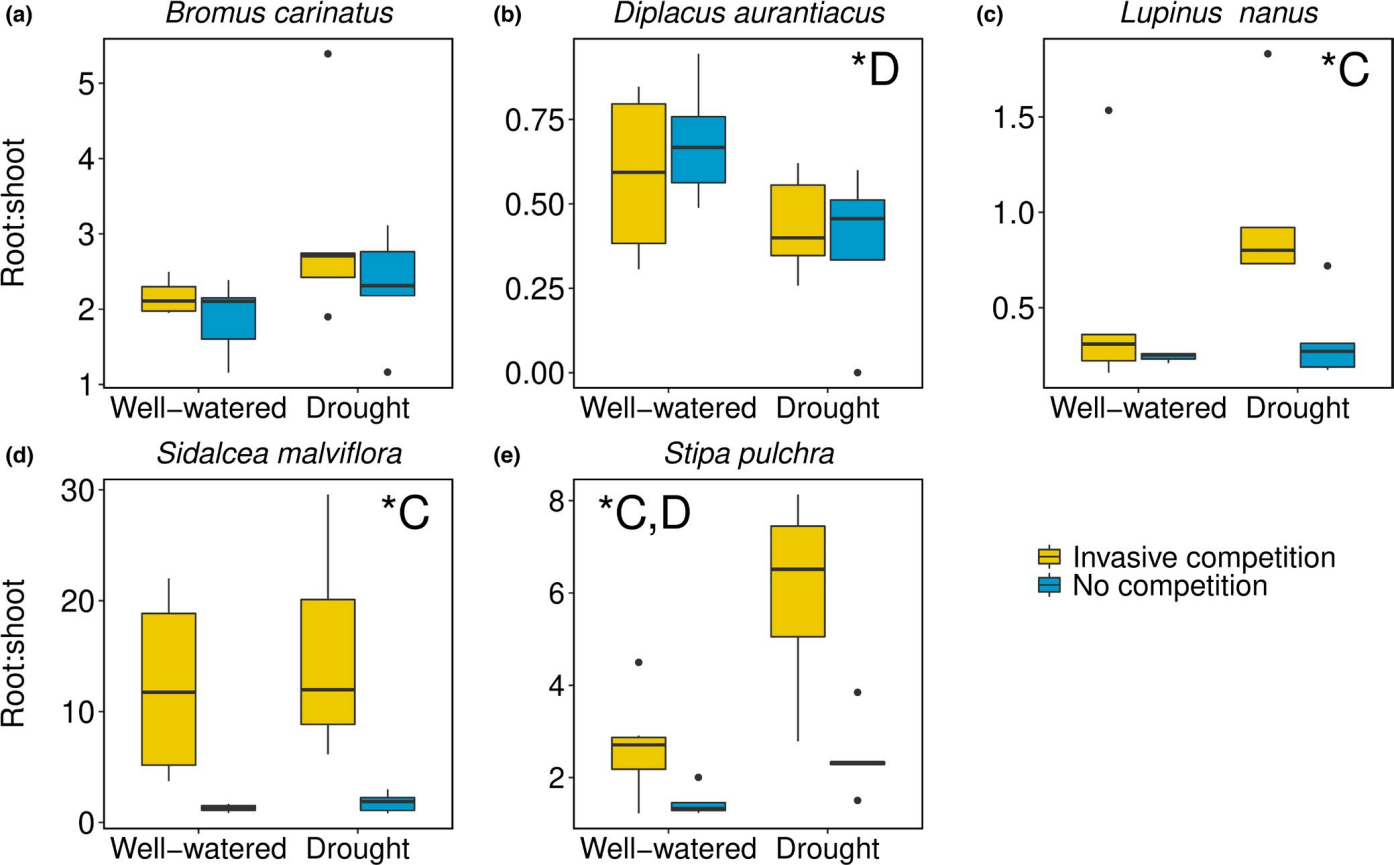
Root:shoot of native species: (a) *Bromus carinatus*, (b) *Diplacus aurantiacus*, (c) *Lupinus nanus*, (d) *Sidalcea malviflora*, and (e) *Stipa pulchra* when experiencing drought and competition from invasive species (yellow) or not (blue). *Denotes significance of C = competition, D = Drought; C, D indicates both competition and drought, but not the interaction (I). The colored bar = interquartile range; the solid line in the bar = median; lines extending out of bar = upper and lower quartile range; and circular points = outliers

The root:shoot of all species, except *Bromus carinatus*, were significantly affected by invasive competition or drought (Figure 1, Table S1). *Diplacus aurantiacus* (*p* = .021) had lower root:shoot in drought, whereas *Lupinus nanus* (*p* = .015) and *Sidalcea malviflora* (*p* = .005) had higher root:shoot in response to invasive competition. *Stipa pulchra* had higher root:shoot from both drought (*p* = .004) and invasive competition (*p* = .001).

### 3.2 Leaf traits

**FIGURE 2:**
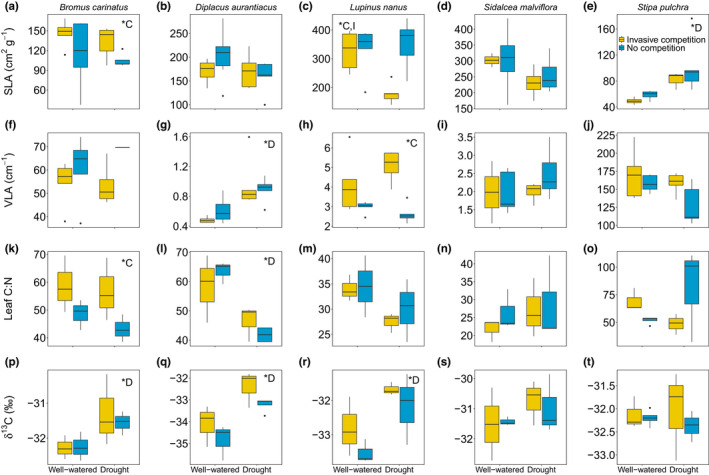
Functional traits (SLA (specific leaf area; a–e), VLA (major vein length per unit area, f–j), leaf C:N (k–o), and δ^13^C (p–t)) for native species experiencing competition from invasive species (yellow) or not (blue). *Denotes significance of C = competition, D = Drought, or I = interaction. The colored bar = interquartile range; the solid line in the bar = median; lines extending out of bar = upper and lower quartile range; and points = outliers

SLA and leaf δ^13^C were the traits most responsive to drought and competition, while leaf lobedness was the least responsive (Figure 2). *Lupinus nanus* had lower SLA (*p* = .014), lower absolute leaf area (*p* = .002), higher VLA (*p* < .001), and higher leaf lobedness (*p* = .002) with invasive competition and higher δ^13^C during drought (*p* = .016). *Diplacus aurantiacus* had smaller leaves (*p* < .001), but higher VLA (*p* < .001), C:N (*p* < .001), and δ^13^C (*p* = .002) in drought. For the grasses, competition increased *B*. *carinatus* SLA (*p* = .047) and C:N (*p* = .041) while drought increased δ^13^C (*p* = .043) and *S*. *pulchra* SLA (*p* = .004). The leaf traits of *S*. *malviflora* were unaffected by drought or competition.

### 3.3 Photosynthetic gas exchange

Midday *A*_net_ and *g*_s_ of *B*. *carinatus*, *D*. *aurantiacus*, and *L*. *nanus* were negatively affected by drought, and further reduced for *L*. *nanus* through an interaction with competition (Table 3, Figure S2F–J). Drought decreased *i*WUE for *D*. *aurantiacus* and *L*. *nanus*, and was further limited by an interaction with competition for *L*. *nanus*. *Diplacus aurantiacus* had an interactive effect, resulting in higher *i*WUE for droughted plants only when experiencing competition (Table 3). Aside from interactions with drought, invasive competition did not affect leaf gas exchange. Midday *A*_net_ (Figure S2A‐E) had a significant and negative reduction over time for all species except *B*. *carinatus*, whereas *g*_s_ decreased over time for all species but *B*. *carinatus* and *S*. *malviflora* (Table 3). *i*WUE had an inverse relationship with time for all species, except for *L*. *nanus*, which had greater *i*WUE over time, and *S*. *malviflora* which had no relationship with time (Figure S2K–O). Midday *i*WUE was positively correlated with leaf δ^13^C of native species (*p* = .016; *R* ^2^ = .51; Figure S3).

**TABLE 3:** Significance (*p*‐values) from midday leaf gas exchange analyses. Bold indicates significant values

Species	Treatment	*A*_net_	*g*	*i*WUE
*Bromus carinatus*	Time	0.301	0.259	**<0.001**
	Well watered × Invasive competition	0.145	0.399	0.597
	Drought × No competition	**0.002**	**<0.001**	0.206
	Drought × Invasive competition	0.561	0.347	0.801
*Diplacus aurantiacus*	Time	**<0.001**	**<0.001**	**0.009**
	Well watered × Invasive competition	0.271	0.593	0.660
	Drought × No competition	**0.016**	**<0.001**	**<0.001**
	Drought × Invasive competition	0.396	0.105	**<0.001**
*Lupinus nanus*	Time	**<0.001**	**0.048**	**<0.001**
	Well watered × Invasive competition	0.114	0.294	0.900
	Drought × No competition	**<0.001**	**<0.001**	**0.032**
	Drought × Invasive competition	**<0.001**	0.126	**0.002**
*Sidalcea malviflora*	Time	**0.016**	0.930	0.428
	Well watered × Invasive competition	0.479	0.343	0.748
	Drought × No competition	0.945	0.116	0.076
	Drought × Invasive competition	0.501	0.490	0.791
*Stipa pulchra*	Time	**<0.001**	**0.011**	**<0.001**
	Well watered × Invasive competition	0.602	0.334	0.907
	Drought × No competition	0.341	0.865	0.943
	Drought × Invasive competition	0.875	0.849	0.845

Note

Treatment effects were compared using generalized linear models with a fixed time effect (based on weekly measurements). *A*_net_ = net CO_2_ assimilation; *g*_s_ = stomatal conductance; *i*WUE = intrinsic water‐use efficiency; *N* = 5 for all groups. All treatments were pooled to test for time effects, significance indicates change over time. Graphical representation (and direction of change) of these findings can be seen in Figure S2.

Invasive competition increased nocturnal respiration for *D*. *aurantiacus* (*p* = .008) and for *S*. *pulchra* facing drought and competition simultaneously (*p* = .010), but no other species (Table S1; Figure S4). Nocturnal respiration was not affected for study species when only facing drought (*p*_all_ > .05). Nocturnal stomatal conductance was negatively affected by drought for *D*. *aurantiacus* (*p* = .040), *L*. *nanus* (*p* < .001), and *S*. *pulchra* (*p* = .004). Nocturnal stomatal conductance of *L*. *nanus* was further reduced by invasive competition in drought conditions (*p* = .012).

### 3.4 Photosynthetic drought loss and recovery

**FIGURE 3:**
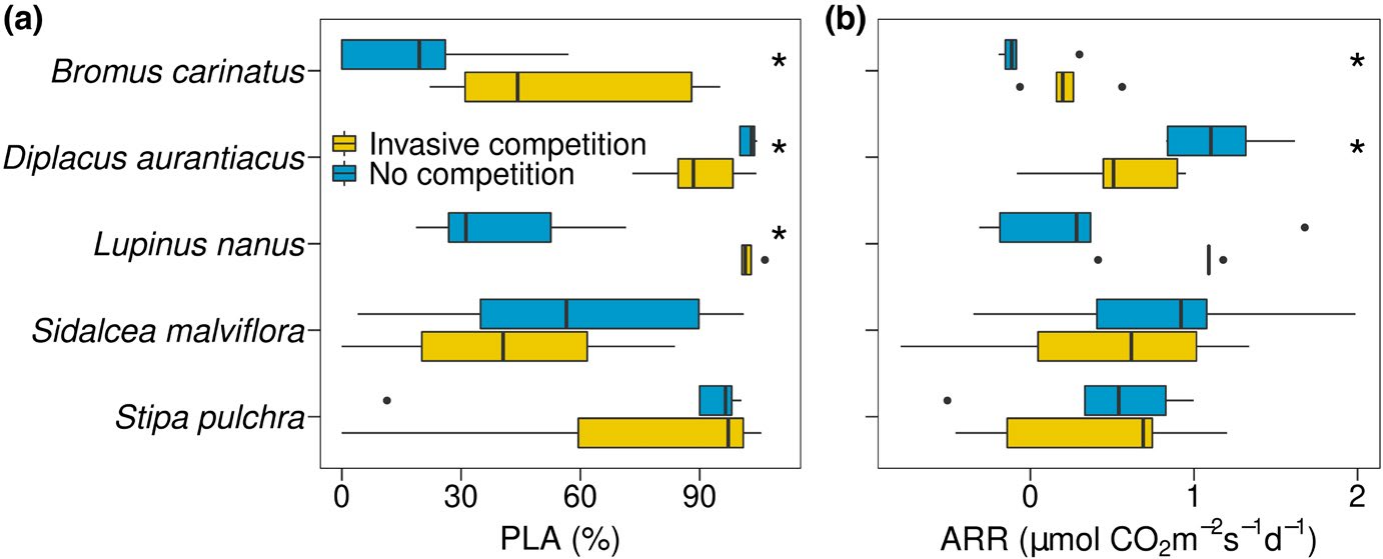
(a) PLA (the percent loss of assimilation) and (b) ARR (the assimilation recovery rates) of native species with competition from invasive species (yellow) or not (blue). *Denotes significant pairwise differences due to competition based on t‐tests. The colored bar = interquartile range; the solid line in the bar = median; lines extending out of bar = upper and lower quartile range; and points = outliers

*Bromus carinatus* (*p* = .046) and *L*. *nanus* (*p* = .001) had greater PLA from drought when experiencing invasive competition, whereas *D*. *aurantiacus* (*p* = .041) had lower drought‐induced photosynthetic loss when in competition (Figure 3a). The recovery rate of assimilation (ARR; Figure 3b) was higher for *B*. *carinatus* (*p* = .039) and lower for *D*. *aurantiacus* (*p* = .019) during competition. Native species percentage recovery of *A*_net_ (PRA) was unaffected by competition (*p*_all_ > .05).

### 3.5 Trade‐offs in growth responses

**FIGURE 4:**
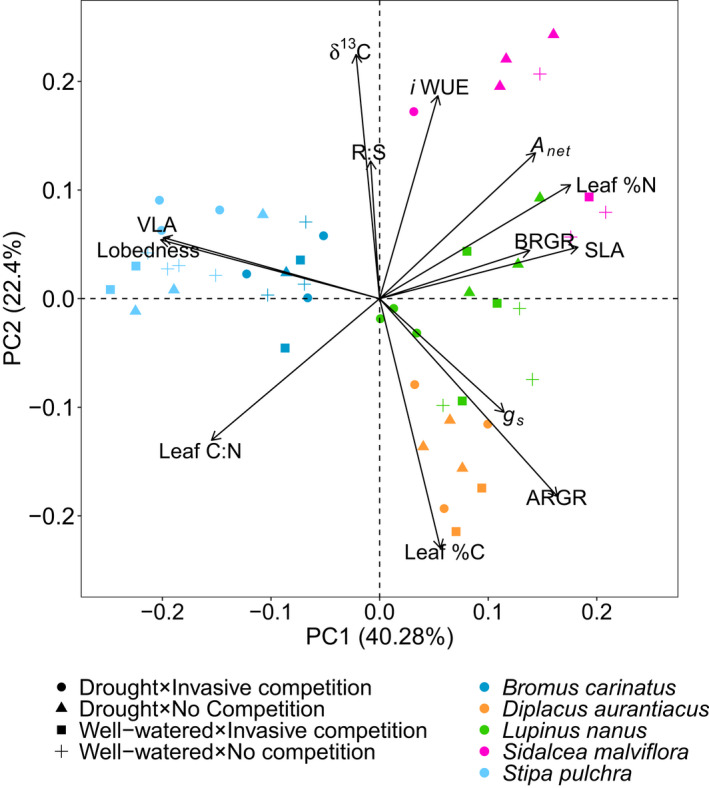
Principal components analysis (PCA) of native species traits experiencing drought and invasive species competition. Vectors indicate where values are highest. Points in the PCA represent the average trait space occupied by the individual plants measured in the experiment and plot within the PCA near vectors they have the greatest values for. Leaf C:N = ratio of leaf carbon:nitrogen; ARGR, aboveground relative growth rate; BRGR, belowground relative growth rate; R:S, dry root:shoot biomass ratio; SLA, specific leaf area; VLA, major vein length per unit area; *A*_net_, net midday CO_2_ assimilation; *g*_s_, net midday stomatal conductance, and *i*WUE, midday intrinsic water‐use efficiency. Units can be found in Table 2

We found that most traits grouped along two principal components (PC) that explained 40.3% and 22.4% of trait variance (Figure 4). Variances were not partitioned by treatments, but instead by species identity. PC1 was related to resource acquisition *versus* conservatism, which Kooyers (2015) related to strategies for drought escape *versus* tolerance (Kooyers, 2015). The acquisition end of the axis was correlated with high SLA, growth rates (ARGR and BRGR), midday *A*_net_, and leaf %N. The resource conservative end of PC1 was related to high leaf C:N, VLA, and leaf lobedness (Table S2). PC2 was driven by trade‐offs related to above‐ *versus* belowground growth allocation. Allocation of resources belowground was associated with high root:shoot, *i*WUE, and δ^13^C, which contrasted with aboveground growth strategies that were correlated with high ARGR and leaf %C (Table S2). Nocturnal leaf respiration, nocturnal *g*_s_, and midday *g*_s_ were not strongly related to either axis.

## 4 DISCUSSION

Most greenhouse‐grown native coastal grassland C_3_ species that we studied exhibited drought‐adapted trait adjustments and a limited amount of adjustments for competition. Our hypothesis that leaf gas exchange would be more affected by drought and less so by competition, and morphological leaf traits more to competition than drought was supported. Moreover, we found evidence (described below) that *D*. *aurantiacus* may experience competitive release during drought. Although it has been shown that drought in California can more negatively affect invasive species than natives, this may be the first evidence to show California species experiencing competitive release in a controlled environment. In support of our predictions and consistent with environmental filter theory, we found trade‐offs between leaf trait conservatism *versus* acquisition. However, we also found trade‐offs related to belowground *versus* aboveground allocation within the multivariate trait space, consistent with optimal partitioning theory.

### 4.1 Invasive competition

According to optimal partitioning theory, increased allocation to roots in response to competition for *L*. *nanus*, *S*. *malviflora*, and *S*. *pulchra* suggests that belowground resources may be more limiting than light or aboveground space for these California coastal grassland species (Bloom et al., 1985; Poorter et al., 2012; Rehling et al., 2021). Aside from biomass allocation, we found certain species adjusted functional traits in response to competition. *Bromus carinatus* exhibited more acquisitive leaf traits (e.g., higher SLA), had more developed root systems to support higher resource needs, and recovered photosynthesis more quickly after drought when undergoing competition from invasives, indicating that this species may be useful for ecological restoration of heavily invaded areas. *Lupinus nanus* had lower leaf area and SLA, but higher VLA and lobedness in competition, which could indicate its sensitivity to competition. A combination of these traits could help increase retention of resources under high demand when contending with competition (Sack & Scoffoni, 2013; Sandel et al., 2021). Higher VLA could facilitate transport of water, photosynthates, and assimilated N (Sack & Scoffoni, 2013), while increased lobedness (Luong et al., 2021) and decreased SLA and leaf area (Pérez‐Harguindeguy et al., 2016) can facilitate reduced transpirational water loss.

### 4.2 Invasion during drought

Although *S*. *pulchra* increased root:shoot allocation in response to drought as predicted by optimal partitioning theory, *D*. *aurantiacus* showed an opposite response (Poorter et al., 2012). But *D*. *aurantiacus* can become woody over time, so investing resources aboveground could provide some degree of drought tolerance (Domec et al., 2017) and enhanced support to compete for light (Sun et al., 2003), and in this regard, responses are consistent with optimal partitioning. Increased δ^13^C and *i*WUE during drought are consistent with upregulated drought tolerance (Lajtha & Marshall, 1994), and consistent with the spectrum of trade‐offs exhibited by PC2 related to above‐ *versus* belowground growth allocation. *Diplacus aurantiacus* and *S*. *pulchra* had higher SLA during drought, which is unexpected based on classic leaf economic spectrum theory (Wright et al., 2004), but consistent with other research for plants in California (Sandel et al., 2021; Welles & Funk, 2021). Higher SLA is related to resource acquisitive strategies (Funk et al., 2008; Wright et al., 2004) and possibly underlies drought escape (Kooyers, 2015), especially for plants in semi‐arid environments. Indeed, other acquisitive traits (*A*_net_, ARGR, BRGR, and %N) responded similarly to SLA in response to factorial drought and competition. Drought tolerance appears to be the strategy used by *D*. *aurantiacus*, as it often actively grows through the summer months and had more resource conservative traits (higher C:N and δ^13^C). The pattern of trait relationships within the resource acquisitive *versus* conservative spectrum is consistent with environmental filter theory, whereas the trade‐offs in above‐ and belowground allocation support optimal partitioning theory (Bloom et al., 1985; Funk et al., 2008).

In general, leaf gas exchange was negatively affected by drought and time, but not competition which supports environmental filter theory's prediction that growth will be more conservative during harsh conditions (Funk et al., 2008). Typically, physiological processes respond in shorter time scales compared to leaf morphology because physiological mechanisms are often molecular (Lambers et al., 2008), which may explain why gas exchange responded to drought. Physiological leaf traits (leaf C:N and δ^13^C) were also primarily affected by drought and not as much by competition. Competition can have mixed effects depending on whether the invader is a stronger above‐ or belowground competitor (Poorter et al., 2012). Similarly, we found that native species exhibited morphological leaf trait (SLA, VLA, and lobedness) adjustments more often to competition, but in certain cases to drought. This response is consistent with optimal partitioning whereby individuals obtain limited aboveground light and space resources (Bloom et al., 1985; Drenovsky et al., 2012). In other instances, morphological traits were responsive to competition, and in a few cases to drought (Poorter et al., 2012). We also note that photosynthesis can decrease as plants age and do not need to compete for space as much as when they are younger (Stromberg et al., 2007).

*Diplacus aurantiacus* showed evidence of competitive release. Because certain invasive species respond more negatively to resource limitation compared to some California natives (Valliere et al., 2019), drought could have facilitated competitive release through increased drought resistance or photosynthetic recovery for natives. *Diplacus aurantiacus* had greater *i*WUE and lower PLA (percent loss of *A*_net_) during drought (indicating higher resistance), but only when competing with invasives. The other native species may not have exhibited competitive release because they were able to adjust their root:shoot or other leaf traits as a result of competition.

## 5 CONCLUSION

The focal native grassland species studied here had diverse responses to drought and invasive competition. Our results provide novel insight into how drought and invasive competition interact to support competitive release for *D*. *aurantiacus* in a controlled environment. Although each manipulation has been tested separately or jointly in the field, there was previously limited work indicating how the factors would interact to influence California plants in a controlled environment. Furthermore, we found morphological traits were primarily affected by invasive competition, whereas physiological traits like photosynthetic gas exchange were primarily affected by drought. Functional traits separated into two axes were related to resource acquisition *versus* conservatism, and aboveground *versus* belowground resource allocation. These relationships are consistent with optimal partitioning and environmental filter theories (Bloom et al., 1985; Funk et al., 2008; Poorter et al., 2012).

Our results have management implications for California grassland restoration and native habitat management. Because certain native species were more resilient or resistant to drought (*B*. *carinatus*, *S*. *malviflora*, and *S*. *pulchra*) and others were more sensitive (*L*. *nanus*), it may be resource effective for restorationists to use drought‐adapted species if planting during extended drought periods, and limit introducing greater species richness to wetter years. Some may also consider using supplemental irrigation if sensitive species must be planted (Stromberg et al., 2007). *Bromus carinatus* exhibited beneficial trait adjustments for higher competitive ability, indicating it may be ideal to use in invaded areas. *Diplacus aurantiacus* showed evidence of competitive release, suggesting that these species will require less invasive species control during drought periods.

## CONFLICT OF INTEREST

Authors declare no conflict of interests.

## AUTHOR CONTRIBUTIONS

**Justin C. Luong:** Conceptualization (equal); Formal analysis (lead); Funding acquisition (supporting); Methodology (supporting); Visualization (lead); Writing – original draft (lead). **Michael E. Loik:** Conceptualization (equal); Formal analysis (supporting); Funding acquisition (lead); Methodology (lead); Visualization (supporting); Writing – original draft (supporting).

## Supporting information

Appendix S1

## Data Availability

Plant trait data were deposited in the TRY‐TRAIT database. Data presented are available (including trait data on TRY‐TRAIT) on PANGAEA Data Publisher for Earth and Environmental Sciences (Luong & Loik, 2022).
