# Supplementary material for: Adjustments in physiological and morphological traits suggest drought‐induced competitive release of some California plants
Source: Ecol Evol. 2022 Apr 1;12(4):e8773. doi: 10.1002/ece3.8773 (PMC8975776; doi:10.1002/ece3.8773)
Supplement: Supplementary file 1 — Appendix S1 [file ECE3-12-e8773-s001.docx]

**Appendix**

**SI Table 1**. Average and standard error of native species traits: aboveground biomass (AGB), belowground biomass (BGB), root:shoot, specific leaf area (SLA), major vein length per unit area (VLA), lobedness, nocturnal leaf gas exchange measurements and the relative distance plasticity index (rdpi; unitless; 0 = no plasticity; 1 = maximum plasticity) of each trait. Species differences were tested using ANOVA; df = 4. Species measurements are pooled from all treatments. Traits were all sampled the week of or during final harvest.

| **Trait** | **F-value** | ***p*** | *Bromus carinatus* | *Diplacus aurantiacus* | *Lupinus nanus* | *Sidalcea malviflora* | *Stipa pulchra* |
| --- | --- | --- | --- | --- | --- | --- | --- |
| AGB (g) | 56.3 | **< 0.001** | 2.214 ± 0.110 | 5.536 ± 0.196 | 4.277 ± 0.358 | 1.781 ± 0.313 | 1.350 ± 0.109 |
| BGB (g) | 18.7 | **< 0.001** | 4.941 ± 0.275 | 3.019 ± 0.238 | 1.656 ± 0.148 | 5.571 ± 0.636 | 3.424 ± 0.282 |
| Root:shoot | 10.9 | **< 0.001** | 2.355 ± 0.205 | 0.525 ± 0.050 | 0.524 ± 0.104 | 7.711 ± 1.92 | 2.983 ± 0.434 |
| Aboveground growth rate (g_•_d^-1^) | 75.0 | **< 0.001** | 0.010 ± 0.001 | 0.053 ± 0.003 | 0.046 ± 0.005 | 0.017 ± 0.004 | -0.008 ± 0.001 |
| Belowground growth rate (g_•_d^-1^) | 30.3 | **< 0.001** | 0.021 ± 0.003 | 0.032 ± 0.004 | 0.019 ± 0.002 | 0.061 ± 0.008 | -0.001 ± 0.002 |
| SLA (cm^2^_•_g^-1^) | 49.5 | **< 0.001** | 125 ± 7.26 | 177 ± 8.94 | 297 ± 21.6 | 269 ± 15.5 | 75.2 ± 6.67 |
| VLA (m^-1^) | 470 | **<0.001** | 57.0 ± 2.45 | 0.73 ± 0.060 | 3.70 ± 0.290 | 2.14 ± 0.13 | 154 ± 5.97 |
| Lobedness | 253 | **< 0.001** | 90.8 ± 2.76 | 10.1 ± 0.31 | 42.1 ± 2.35 | 21.8 ± 3.79 | 297 ± 15.1 |
| Leaf C (%) | 71.6 | **< 0.001** | 39.8 ± 0.267 | 49.4 ± 0.161 | 42.5 ± 0.172 | 35.5 ± 0.550 | 38.6 ± 0.022 |
| Leaf N (%) | 31.3 | **< 0.001** | 0.908 ± 0.029 | 1.17 ± 0.048 | 1.62 ± 0.059 | 1.68 ± 0.081 | 0.808 ± 0.064 |
| Leaf C:N | 17.7 | **< 0.001** | 51.9 ± 2.18 | 51.7 ± 2.37 | 31.5 ± 1.11 | 26.0 ± 1.58 | 61.9 ± 5.00 |
| Leaf δ^13^C (‰) | 22.5 | **< 0.001** | -31.8 ± 0.152 | -33.6 ± 0.245 | -32.5 ± 0.211 | -31.2 ± 0.177 | -32.2 ± 0.102 |
| Leaf δ^15^N (‰) | 33.9 | **< 0.001** | -2.26 ± 0.203 | 2.60 ± 0.380 | 2.76 ± 0.147 | 3.50 ± 0.475 | -0.735 ± 0.407 |

**SI Table 1 continued – page 2**.

| **Trait** | **F-value** | ***p*** | *Bromus carinatus* | *Diplacus aurantiacus* | *Lupinus nanus* | *Sidalcea malviflora* | *Stipa pulchra* |
| --- | --- | --- | --- | --- | --- | --- | --- |
| SLA rdpi | 12.1 | **< 0.001** | 0.114 ± 0.056 | -0.179 ± 0.039 | -0.136 ± 0.078 | -0.160 ± 0.049 | 0.375 ± 0.147 |
| VLA rdpi | 5.50 | **< 0.001** | -0.084 ± 0.040 | 0.279 ± 0.129 | 0.328 ± 0.122 | 0.126 ± 0.095 | -0.036 ± 0.051 |
| Lobedness rdpi | 2.18 | 0.083 | -0.035 ± 0.031 | 0.034 ± 0.042 | 0.102 ± 0.072 | 0.518 ± 0.368 | 0.023 ± 0.065 |
| Leaf % C rdpi | 5.69 | **0.001** | 0.033 ± 0.014 | -0.001 ± 0.006 | 0.051 ± 0.004 | -0.062 ± 0.030 | -0.040 ± 0.009 |
| Leaf % N rdpi | 5.77 | **0.001** | -0.036 ± 0.041 | 0.324 ± 0.055 | 0.171 ± 0.045 | -0.038 ± 0.061 | -0.148 ± 0.099 |
| Leaf C:N rdpi | 4.59 | **0.005** | 0.088 ± 0.058 | -0.241 ± 0.039 | -0.116 ± 0.034 | -0.019 ± 0.087 | 0.280 ± 0.133 |
| Leaf δ^13^C rdpi | 6.21 | **< 0.001** | -0.017 ± 0.006 | -0.046 ± 0.007 | -0.039 ± 0.006 | -0.011 ± 0.009 | -0.001 ± 0.005 |
| Leaf δ^15^N rdpi | 5.19 | **0.002** | -0.166 ± 0.094 | 0.153 ± 0.206 | 0.205 ± 0.063 | -0.013 ± 0.169 | -0.944 ± 0.231 |
| *C. pycnocephalus* biomass (g) | 16.5 | **< 0.001** | 0.045 ± 0.006 | 0.079 ± 0.028 | 0.431 ± 0.082 | 0.760 ± 0.181 | 0.058 ± 0.009 |
| *F. bromoides* biomass (g) | 48.7 | **< 0.001** | 0.329 ± 0.027 | 0.316 ± 0.025 | 0.817 ± 0.070 | 1.48 ± 0.133 | 0.531 ± 0.032 |
| *G. dissectum* biomass (g) | 15.5 | **< 0.001** | 0.223 ± 0.013 | 0.181 ± 0.021 | 0.421 ± 0.058 | 0.736 ± 0.119 | 0.258 ± 0.023 |
| *M. polymorpha* biomass (g) | 3.41 | **0.017** | 0.002 ± 0.001 | 0.003 ± 0.001 | 0.003 ± 0.002 | 0.010 ± 0.002 | 0.008 ± 0.003 |
| *R. sativus* biomass (g) | 16.3 | **< 0.001** | 0.064 ± 0.009 | 0.109 ± 0.028 | 0.477 ± 0.083 | 0.724 ± 0.194 | 0.062 ± 0.012 |

**SI Table 1 continued – page 3**.

| **Trait** | **F-value** | ***p*** | *Bromus carinatus* | *Diplacus aurantiacus* | *Lupinus nanus* | *Sidalcea malviflora* | *Stipa pulchra* |
| --- | --- | --- | --- | --- | --- | --- | --- |
| Percent loss of *A_net_*(PLA) | 5.95 | **< 0.001** | 38.3 ± 10.5 | 96.0 ± 3.34 | 71.3 ± 11.3 | 50.1 ± 12.2 | 76.0 ± 12.4 |
| Percent recovery of *A_net_*(PRA) | 2.86 | **0.036** | 87.6 ± 11.1 | 53.1 ± 6.92 | 66.4 ± 10.9 | 90.7 ± 7.50 | 104 ± 18.4 |
| Recovery rate of *A_net_* (µmol CO_2_ m^-2^s^-1^ d^-1^; ARR) | 2.49 | 0.059 | 0.087 ± 0.078 | 0.843 ± 0.150 | 0.669 ± 0.206 | 0.647 ± 0.284 | 0.423 ± 0.190 |
| Last day midday *A_net_* (µmol CO_2_ m^-2^s^-1^) | 10.2 | **< 0.001** | 4.956 ± 0.472 | 2.486 ± 0.542 | 6.371 ± 1.015 | 9.597 ± 1.105 | 4.468 ± 0.803 |
| Last day midday *g_s_* (mol H_2_O m^-2^s^-1^) | 5.68 | **< 0.001** | 0.127 ± 0.012 | 0.170 ± 0.038 | 0.140 ± 0.026 | 0.328 ± 0.061 | 0.130 ± 0.022 |
| Last day midday intrinsic Water-use efficiency (µmol CO_2_ mol H_2_O^-1^) | 2.90 | **0.026** | 39.31 ± 2.284 | 25.4 ± 8.421 | 42.0 ± 6.530 | 50.4 ± 10.10 | 22.8 ± 4.097 |
| Last day dark-hours respiration rate (µmol CO_2_ m^-2^s^-1^) | 5.04 | **< 0.001** | 0.513 ± 0.027 | 0.483 ± 0.070 | 0.744 ± 0.100 | 0.514 ± 0.050 | 0.370 ± 0.049 |
| Last day dark-hours *g_s_* (mol H_2_O m^-2^s^-1^) | 8.37 | **< 0.001** | 0.012 ± 0.003 | 0.057 ± 0.023 | 0.013 ± 0.002 | 0.001 ± 0.0004 | 0.058 ± 0.007 |

**SI Table 2.** Correlations between traits and two main principal components. The higher the absolute value, the more correlated the trait is to the principal component. Traits with >50% correlation with PC axes are bolded.

| Trait | PC1 (40.3%) | PC2 (22.4%) |
| --- | --- | --- |
| SLA (cm^2^g^-1^) | **0.800** | 0.154 |
| VLA (cm^-1^) | **-0.904** | 0.186 |
| Lobedness (unitless) | **-0.885** | 0.171 |
| Root:shoot (unitless) | -0.042 | **0.592** |
| ARGR (g•d^-1^) | **0.706** | **-0.592** |
| BRGR (g•d^-1^) | **0.661** | 0.194 |
| Leaf C:N | **-0.688** | -0.414 |
| Leaf %C | 0.276 | **-0.834** |
| Leaf %N | **0.776** | 0.319 |
| Leaf δ^13^C (‰) | -0.099 | **0.767** |
| CO_2_ Assimilation (µmol CO_2_ m^-2^s^-1^) | **0.607** | 0.432 |
| Stomatal conductance (mol H_2_O m^-2^s^-1^) | 0.462 | -0.301 |
| *i*WUE (µmol CO_2_ mol H_2_O^-1^) | 0.253 | **0.635** |


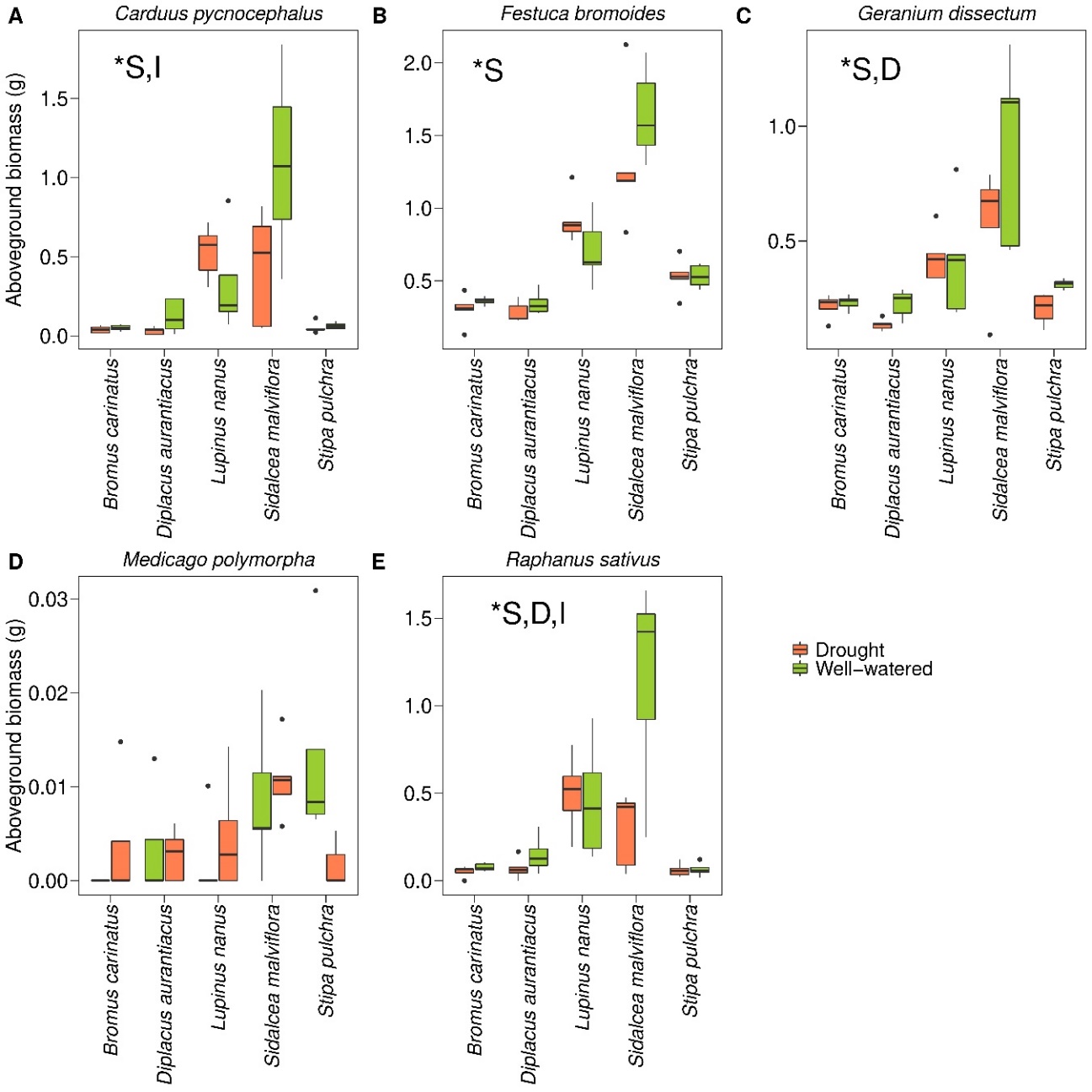


**SI Figure 1**. Total aboveground biomass production of invasive species per pot when in competition with native competitors: (**A**) *Carduus pycnocephalus*, (**B**) *Festuca bromoides*, (**C**) *Geranium dissectum*, (**D**) *Medicago polymorpha*, and (**E**) *Raphanus sativus*. *denotes significance of S = native competition, D = Drought, or I = interaction. Orange bars represent plants experiencing drought and green represents well-watered conditions. The colored bar = interquartile range, the solid line in the bar = median; lines extending out of bar = upper and lower quartile range; points = outliers.


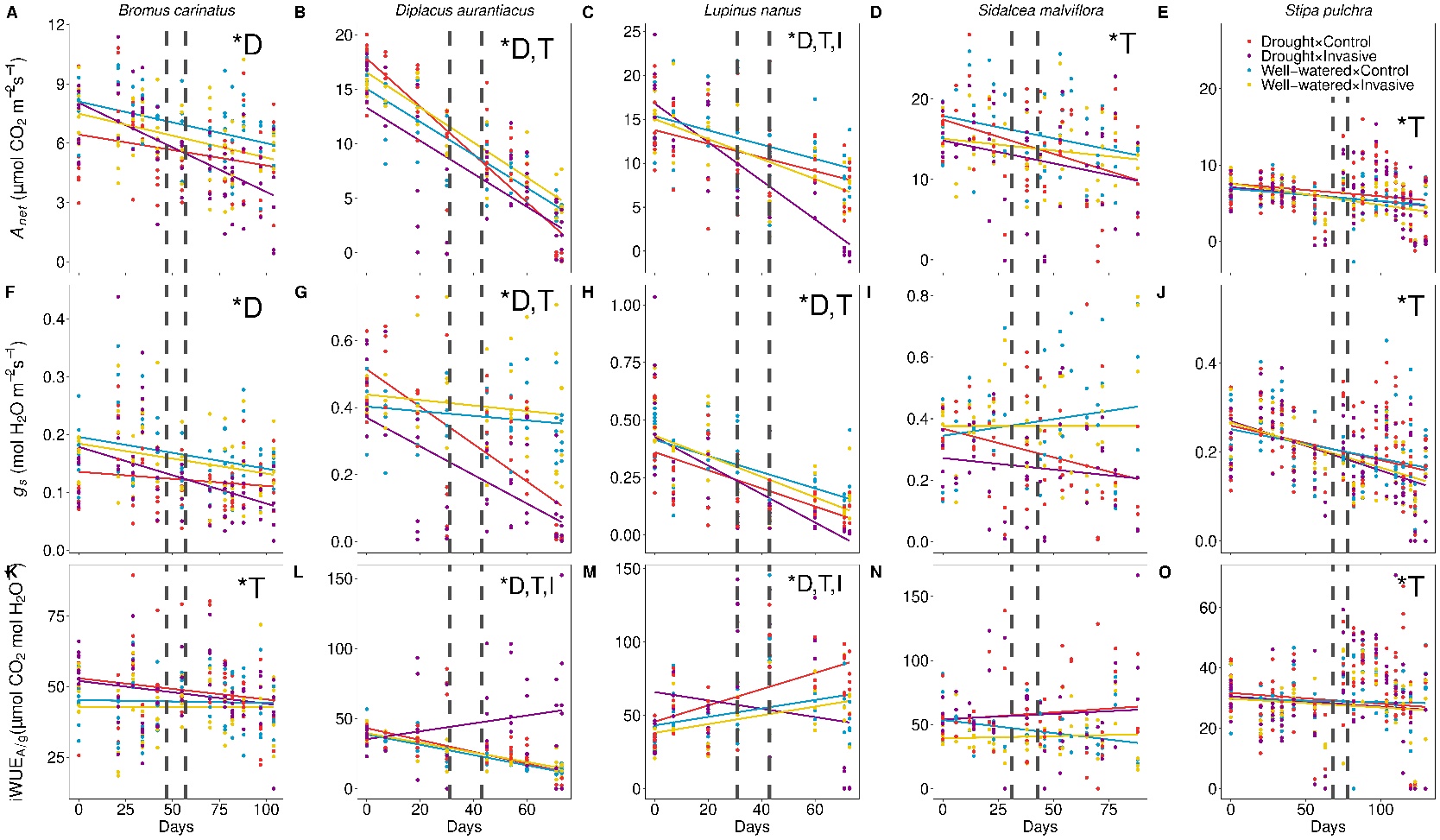


**SI Figure 2**. Fixed effects linear models for carbon assimilation rates [*A_net_* (µmol CO_2_ m^-2^s^-1^)] (**A-E**), stomatal conductance [*g_s_* (mol H_2_O m^-2^s^-1^)] (**F-J**) and intrinsic Water-use efficiency [*i*WUE (µmol CO_2_ mol H_2_O^-1^)] (**K-O**) for native species. Leaf gas-exchange measurements occurred at midday at least once every week. Lines were fitted with linear regressions. *denotes significance of C = competition (from experimental invasion), D = Drought, T = Time, or I = interaction; commas indicates multiple factors, but not the interaction. Blue = well-watered × no competition; Red = drought × no competition; Yellow = well-watered × invasive competition; Purple = drought × invasive competition. Space before the first dashed line is the first drought period; and after the dashed line is the second drought period.


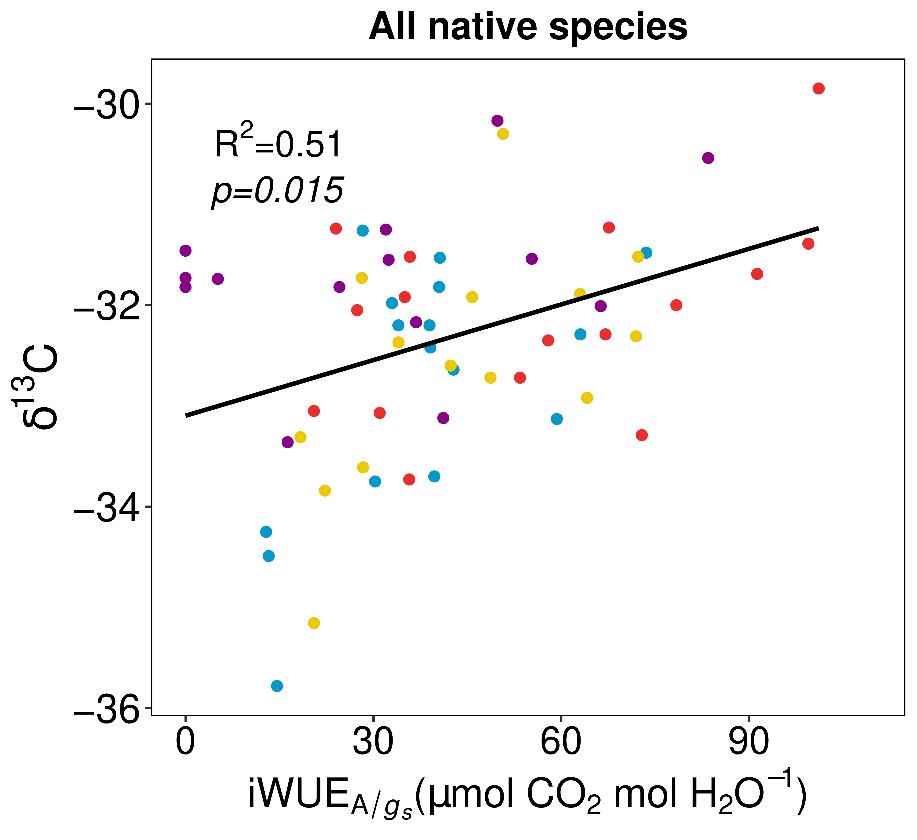


**SI Figure 3**. Relationship between pooled native species leaf δ^13^C (‰) and *i*WUE (intrinsic Water-use efficiency; µmol CO_2_ mol H_2_O^-1^) measured as a ratio of CO_2_ assimilation to stomatal conductance. Blue = well-watered × no competition; Red = drought × no competition; Yellow = well-watered × invasive competition; Purple = drought × invasive competition.

**
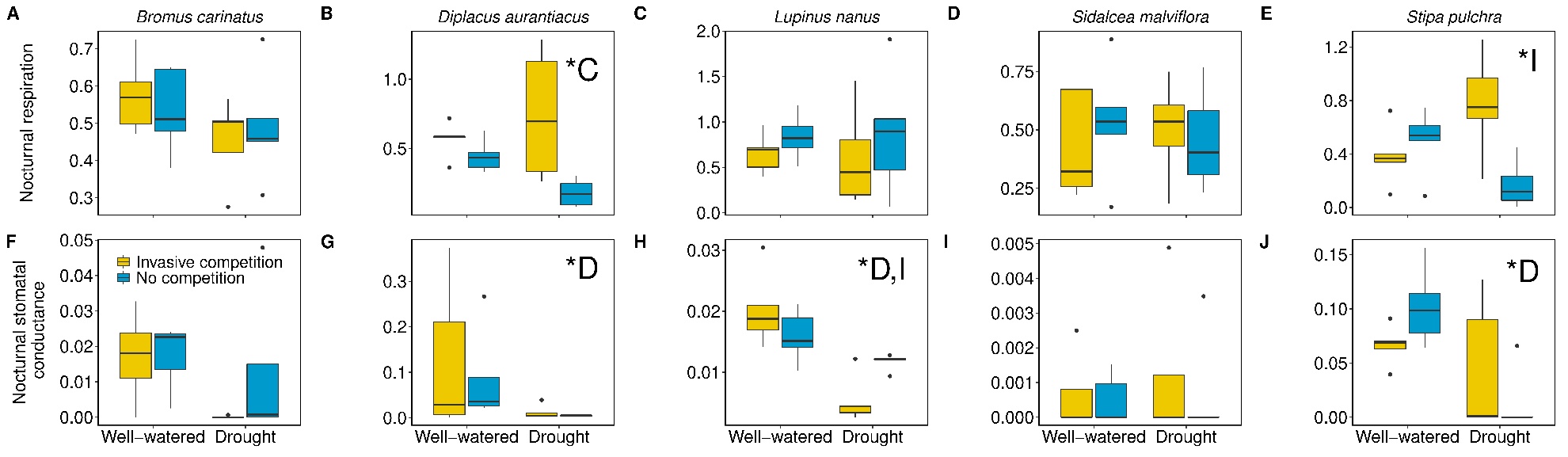
**

**SI Figure 4**. Nocturnal respiration (µmol CO_2_ m^-2^s^-1^; **A-E**) and nocturnal stomatal conductance (mol H_2_O m^-2^s^-1^_;_ **F-J**) for plants experiencing drought with competition from invasive species (yellow) or not (blue). *denotes significance of C = competition, D = Drought, or I = interaction. The colored bar = interquartile range, the solid line in the bar = median; lines extending out of bar = upper and lower quartile range; points = outliers.
